# Enhancement of growth performance and health status in heat-stressed growing rabbits using alpha-lipoic acid-loaded chitosan nanoparticles

**DOI:** 10.5713/ab.25.0456

**Published:** 2025-08-25

**Authors:** Ali Ali El-Raghi, Huda A. Al Doghaither, Ayat B. Al-Ghafari, Mahmoud A. E. Hassan, Reham. A. Elbhnsawy, Naglaa A. Gomaa, Ekramy M. Elmorsy

**Affiliations:** 1 Department of Animal, Poultry, and Fish Production, Faculty of Agriculture, Damietta University, Damietta, Egypt; 2 Department of Biochemistry, Faculty of Science, King Abdulaziz University, Jeddah, Saudi Arabia; 3 Experimental Biochemistry Unit, King Fahd Medical Research Center, King Abdulaziz University, Jeddah, Saudi Arabia; 4 Animal Production Research Institute (APRI), Agriculture Research Center, Ministry of Agriculture, Giza, Egypt; 5 Department of Poultry and Rabbit Diseases, Veterinary Teaching Hospital, Faculty of Veterinary Medicine, Mansoura University, Mansoura, Egypt; 6 Department of Clinical Sciences, College of Veterinary Medicine, King Faisal University, Al-Ahsa, Saudi Arabia; 7 Department of Animal Medicine, Faculty of Veterinary Medicine, Kafrelsheikh University, Kafrelsheikh, Egypt; 8 Center for Health Research, Northern Border University, Arar, Saudi Arabia

**Keywords:** Alpha-lipoic Acid-loaded Chitosan Nanoparticles, Health Status, Heat Stress, Growth, Rabbits

## Abstract

**Objective:**

This study investigates the effect of alpha-lipoic acid-loaded chitosan nanoparticles (ALA-CHNPs) on growth performance, feed utilization, and health in heat-stressed growing rabbits.

**Methods:**

A total of 125 healthy, 5-week-old rabbits were randomly assigned to five groups of 25. One group served as a thermoneutral control, while the other four groups were subjected to heat stress and received diets supplemented with 0, 100, 200, or 400 mg of ALA-CHNPs per kilogram, respectively.

**Results:**

Dietary supplementation resulted in significant improvements in growth performance, feed conversion ratio, physiological responses, liver weight, and dressing percentage. Supplementation with ALA-CHNPs resulted in a linear reduction in liver enzymes activities, as well as in the levels of total bilirubin, hydrogen peroxide, glutathione, catalase, superoxide dismutase, immunoglobulin G, lysozyme activity, interleukin (IL)-6 and IL-10. No significant differences were observed between the ALA-CHNPs200 and ALA-CHNPs400 groups (p>0.05). The levels of blood serum total protein, albumin, globulin, total antioxidant capacity, glutathione peroxidase activity, immunoglobulin A and M, and nitric oxide showed a quadratic increase, reaching a peak at ALA-CHNPs doses ranging from 250–300 mg/kg diet. Conversely, the concentrations of triglycerides, cholesterol, glucose, malondialdehyde, interferon-gamma, tumor necrosis factor-alpha, and nuclear factor kappa B exhibited a quadratic decrease, with optimal reductions observed at doses between 250–300 mg/kg diet. Supplementation with ALA-CHNPs reduced liver damage caused by heat stress, restoring normal hepatic morphology. The hydrophobic interactions of ALA with antioxidant enzymes and cytokines contributed to the reduction of heat stress-induced oxidative stress.

**Conclusion:**

The inclusion of 250–300 mg ALA-CHNPs/kg diet enhanced growth performance, redox balance, immune function, and inflammatory response in fattened rabbits raised during the summer season.

## INTRODUCTION

Heat stress (HS), intensified by prolonged heat waves and global warming, presents a major challenge to farm animals, with rabbits being particularly at risk [1]. Their limited number of sweat glands and thick fur make effective heat dissipation difficult, increasing their vulnerability [2]. Rabbits have a narrow thermo neutral range of 15°C–21°C [3], making them highly susceptible to HS, especially in tropical and subtropical climates. When exposed to temperatures above this range, especially during the summer, their skin and body temperatures rise, promoting excessive free radical generation. This leads to oxidative stress (OS), marked by lipid peroxidation and damage to cellular proteins, lipids, and DNA, ultimately impairing feed utilization and growth performance [4,5]. Recent studies have indicated that prolonged exposure to HS in rabbits leads to increased levels of pro-inflammatory cytokines and elevated non-specific immune responses [5,6]. With the rise of intensive industrial farming practices and the ongoing impacts of global warming, the severity of HS in animals is escalating, presenting a major challenge to the rabbit industry. Consequently, exploring effective alternative feeding strategies to substitute antibiotics and boost heat tolerance in rabbits has become increasingly essential. Natural feed additives provide a sustainable, safe, practical, and cost-effective approach to mitigate the harmful impacts of HS on rabbits’ growth, antioxidant capacity, immunity, and inflammation. Recent studies highlight the importance of nutritional interventions in strengthening animals’ resilience to HS and promoting their overall health and productivity [7].

Alpha-lipoic acid (ALA) is a potent, versatile antioxidant. It possesses metal-chelating abilities, aids in the regeneration of endogenous antioxidants such as glutathione, vitamins C and E, and helps neutralize reactive oxygen species [8]. ALA also aids in carbohydrate metabolism and energy production by serving as a cofactor for essential enzymes [9]. Furthermore, it enhances antioxidant levels, improves lipid profiles, supports liver function, regulates immune gene expression, and positively impacts meat quality and growth performance in broilers [8]. Although ALA offers several benefits, its poor water solubility and sensitivity to low pH, light, and heat hinder its bioavailability and stability [10]. Therefore, it is essential to develop advanced technologies that can improve ALA’s bioavailability and stability for its effective application as a pharmaceutical agent and dietary supplement. Over the past two decades, nanoparticles (NPs) have garnered considerable interest in drug delivery systems for their ability to enhance drug solubility, efficacy, stability, and minimize toxicity [11]. Typically smaller than 300 nm, NPs provide controlled release and can encapsulate both hydrophobic and hydrophilic drugs [12]. Polymeric NPs, particularly those derived from chitosan, a natural polymer, show great potential for targeted delivery of complex molecules like RNA. Chitosan NPs are biodegradable, biocompatible, and exhibit low toxicity. Their effectiveness is affected by factors such as surface characteristics and particle size [5,11]. This study investigates the use of ALA-loaded chitosan nanoparticles (ALA-CHNPs) as an innovative delivery system to improve the stability, solubility, and efficacy of ALA. Although previous studies have highlighted the wide range of biomedical applications for ALA [8], the potential benefits of incorporating ALA-CHNPs into the diets of growing rabbits under serve HS conditions have not yet been explored. Therefore, this study aims to be the first to evaluate the impact of ALA-CHNPs on growth performance, feed efficiency, carcass traits, meat quality, redox status, immune function, and inflammation in growing rabbits exposed to severe HS conditions.

## MATERIALS AND METHODS

The experiment was carried out during the summer season (July and August) on a private farm located in Mansoura city, Dakahlia Governorate, Egypt. Organic ALA was obtained from AB Chem Company, Mansoura city, Egypt.

### Molecular docking

Molecular docking studies evaluated ALA interactions with key antioxidant and inflammatory proteins involved in HS response in developing rabbits. ALA’s 3D structure was optimized from PubChem data, while protein targets like catalase (CAT), superoxide dismutase (SOD), glutathione peroxidase (GPx), interlukin-4 (IL-4), IL-6, tumor necrosis factor alpha (TNF-α), interferon gamma (IFN-γ), lysozyme (Lyz), and nuclear factor-kappa B (NF-κB) were obtained from the RCSB Protein Data Bank. Docking was performed using the EADock DSS algorithm on SwissDock with a blind docking approach. Binding affinity, hydrogen bonds, hydrophobic, and ionic interactions were analyzed, focusing on key active sites. The most favorable conformations were selected for detailed analysis.

### Preparation of alpha-lipoic acid-loaded chitosan nanoparticles

ALA-CHNPs were prepared by dissolving chitosan in 1% acetic acid (2 mg/mL), adding 1% Tween 80, sonicating for 15 minutes, and adjusting the pH to 5 with 2N NaOH. ALA at different levels (0–3,000 μg/mL) was added to the chitosan solution and sonicated for 5 minutes. Sodium tripolyphosphate, prepared in deionized water at a chitosan-to-sodium tripolyphosphate weight ratio of 5:1, was then added dropwise under continuous stirring. The resulting dispersions were stabilized by magnetic stirring for 60 minutes to allow complete cross-linking and stabilization. The ALA-CHNPs were initially frozen at −80°C, followed by lyophilization under vacuum conditions to obtain the dry NP powder. The polydispersity index (PDI), zeta potential, and hydrodynamic mean diameter of ALA-CHNPs were measured using a Zetasizer NanoZS, with zeta potential assessed in triplicate and distilled water used for Z-average dilution. Surface morphology was analyzed by transmission electron microscopy (TEM; TEM-JEOL 2100) at 160 kV. It is worth noting that the chitosan used in this study possessed an approximate deacetylation degree of 85% and a medium molecular weight (190–310 kDa) as reported by the manufacturer (e.g., Sigma-Aldrich). These characteristics are known to influence drug encapsulation capabilities and the formulation’s physicochemical stability.

### Determination of loading efficiency and loading content

The loading efficiency (LE) and loading content (LC) of ALA in the ALA-CHNPs were determined by measuring the concentration of free ALA in the supernatant after centrifugation, using a UV-Visible spectrophotometer at 334 nm according to the following equations:


(1) LE (%)=(Weight of ALA in NPs/Weight of ALA initially added)×100

(2) LC (%)=(Weight of ALA in NPs/Total weight of NPs)×100

### Animals, experimental design, and diets

All animal procedures were conducted in accordance with the institutional guidelines for the care and use of laboratory animals and complied with internationally recognized ethical standards, including the EU Directive 2010/63/EU on the protection of animals used for scientific purposes. The experimental protocol was reviewed and ethically approved by the researchers’ institution prior to the commencement of the study. A total of 125 male APRI rabbits, five weeks old with an initial body weight of 661.43±7.92 g, were randomly allocated into five experimental groups, with 25 rabbits in each group. The thermoneutral control group was fed a basal diet and maintained under optimal environmental conditions for rabbits, including a relative humidity (RH) of 55%–65%, temperature range of 18°C–22°C, proper ventilation, and complete avoidance of HS. The HS control group was fed only the basal diet, whereas the other three heat-stressed groups received the basal diet supplemented with ALA-CHNPs at levels of 100 mg/kg (ALA-CHNPs100), 200 mg/kg (ALA-CHNPs200), and 400 mg/kg (ALA-CHNPs400), respectively. These concentrations represent the total amount of ALA-CHNPs, including both the chitosan polymer matrix and the entrapped ALA. The animals were housed in individual wire cages measuring 40 cm in height, 50 cm in length, and 30 cm in width. The experiment lasted for eight weeks, ending at 13 weeks of age, with water and feed available *ad libitum*. The animals’ health was closely monitored throughout the study period. All animals were kept under uniform managerial, environmental, and hygienic conditions, and were fed according to the National Research Council (NRC) guidelines. The ingredients and chemical composition of the basal diet are provided in Table 1.

### Climatic condition

In the rabbitry, ambient air temperature and RH (%) were measured daily with an automatic thermo-hygrometer (Dostmann). The Temperature-Humidity Index (THI) was calculated using the formula of Marai et al [3].


(3) $$\text{THI}=\text{dp}-\left[\left(0.31-0.31\left(\frac{\text{RH}}{100}\right)\right)\times (\text{dp}-14.4)\right]$$

Where dp represents the dry bulb temperature in Celsius (°C). The THI values were categorized as follows: no HS<27.8; moderate HS, 27.8 to 28.9; severe HS, 29.0 to 30.0; and very severe HS>30.0.

### Assessing growth indices, carcass traits and chemical composition

Live body weight (LBW) and Feed intake were measured biweekly throughout the study period for each rabbit. This data was used to calculate the feed conversion ratio (FCR), average daily weight gain (ADG), and performance index (PI) using the following formulas:


(4) FCR=Grams of feed/Gram of body weight gain

(5) ADG (g)=(Final body weight-Initial body weight)/Fattening period

(6) PI (%)=(FBW [kg]×100)/FCR.

After 8 weeks of the feeding trial, ten rabbits from each group were randomly selected and fasted for 12 hours, and slaughtered following the Islamic method, which involves severing the two jugular veins without the use of anesthesia. Non-edible parts, including the pelt, tail, and viscera, were removed. The weights of the kidneys, spleen, heart, liver, lungs, cecum, and intestines were recorded and expressed as a percentage of the LBW. The dressing percentage was calculated using the following equation:


(7) The dressing percentage=(Hot carcass weight/Live body weight)×100

Meat samples were taken from the hind leg muscle and chemically analyzed for crude protein (CP), ash, and ether extract content according to the AOAC International [13] method. CP, fat, and ash were determined by drying 100 g of meat. CP was measured using the Kjeldahl method with a Buchi analyzer (Centec Automatika), fat was assessed using the Soxhlet method (Thermo Fisher Scientific), and ash content was determined by incinerating samples at 550°C, following Gál et al [14].

### Serum biochemical and antioxidants indices

During the slaughtering, ten blood samples were obtained from slaughtered rabbits using sterile tubes without heparin. After allowing the blood to coagulate at room temperature for 30 minutes, the samples were centrifuged at 1,600×g for 15 minutes to separate the serum. The obtained serum was stored at −20°C for subsequent biochemical analysis. Levels of total protein, globulin, total triglycerides (TG), total cholesterol (TC), and glucose (Glu) were determined using colorimetric assay kits (Biosino Bio-technology and Science), in accordance with the manufacturer’s guidelines. Albumin levels were determined by subtracting globulin levels from the corresponding total protein levels. The activities of aspartate aminotransferase (AST), alanine aminotransferase (ALT), and lactate dehydrogenase (LDH) enzymes were measured using specialized commercial kits provided by Biodiagnostic, following the manufacturer’s guidelines. Serum levels of total antioxidant capacity (TAC), SOD, CAT, glutathione peroxidase (GSH-Px), glutathione (GSH), and malondialdehyde (MDA) were determined using specific commercial kits (BioMérieux), in accordance with the manufacturer’s guidelines. The corresponding kit codes used were TAC-2513, SOD-2521, CAT-2517, GSH-Px-2524, GSH-260, and MDA-2529, respectively. The Phenol Red method was employed to colorimetrically measure hydrogen peroxide (H_2_O_2_) concentration at 510 nm using a commercial kit (HP 25; Bio-Diagnostic).

### Immune and pro-inflammatory cytokines

At the end of the experimental period, serum levels of immunoglobulins (IgG, IgM, and IgA) were measured using a SimpleStep ELISA kit according to the manufacturer’s instructions. Lyz activity was assessed using a Lyz activity assay kit (ID: LS-K52-100; LifeSpan BioSciences), following the method described by Toro et al [15]. Cytokine levels including IFN-γ (Cat. No.: E-EL-RB0679), TNF-α (Cat. No.: E-EL-RB0011), interleukin 6 (IL-6; Cat. No.: E-EL-RB0014), interleukin 10 (IL-10; Cat. No.: E-CL-R0016) were determined using ELISA kits from Elabscience. Serum nitric oxide (NO) levels were measured using a colorimetric NO Assay Kit (ab65328; wavelength: 540 nm), following the protocol of Csonka et al [16]. NF-κB (Cat. No.: MBS285445) levels were evaluated using an ELISA kit from Beyotime Biotechnology, in accordance with the manufacturer’s instructions.

### Histology

Following slaughter, liver samples were collected from all experimental groups. The samples were fixed in 10% neutral buffered formalin, dehydrated through a graded series of ethyl alcohol, and cleared in two changes of xylene. Subsequently, they were embedded in paraffin and sectioned at a thickness of 4 μm using a microtome (Leica RM 2155). Tissue sections were stained with eosin and hematoxylin. High-resolution photomicrographs were captured using a Leica DM500 digital microscope integrated with an EC3 digital camera. In this study, histopathological assessments of liver tissues were performed on three rabbits. Each slide contained three tissue sections, with four microscopic fields per section examined at 400× magnification, yielding a total of 12 fields per organ. Lesions were semi-quantitatively scored using a standardized scale (0 = none, 1 = mild, 2 = moderate, 3 = severe), as detailed in Table 2. For each rabbit, the mean lesion score was calculated by averaging the scores from all examined fields in each organ.

### Transmission electron microscope

Liver samples were first fixed in cold 2.5% glutaraldehyde prepared in 0.1 M phosphate buffer for 24 hours at 4°C, then rinsed and post-fixed for 2 hours in 1% osmium tetroxide. The specimens were then dehydrated through a graded ethanol series (50%, 70%, 90%, 95%, and 100%), rinsed with acetone, and embedded in epoxy resin. Ultrathin sections (60–70 nm) were prepared using an ultra-microtome, then stained with uranyl acetate and lead citrate. These sections were subsequently examined with a TEM (TEM-JEOL 2100) at 160 kV.

### Statistical analysis

Data were recorded in Excel, and normality was tested using the Shapiro-Wilk test. Statistical analysis was conducted using the GLM procedure (SAS, 2012) to assess growth parameters, feed utilization, carcass characteristics, meat quality, and blood serum parameters. When treatment effects were statistically significant (p<0.05), the Tukey test was applied to identify differences between means (p<0.05). Polynomial regression analysis was performed to examine the relationships between different levels of ALA-CHNPs (0, 100, 200, and 400 mg/kg diet) and the various parameters. All curves were generated using GraphPad Prism software (ver. 8.0; GraphPad). Multivariable analyses were conducted via SRplot-Science and research online plot (https://www.bioinformatics.com.cn/)

## RESULTS

### Molecular docking

**Figure 1:**
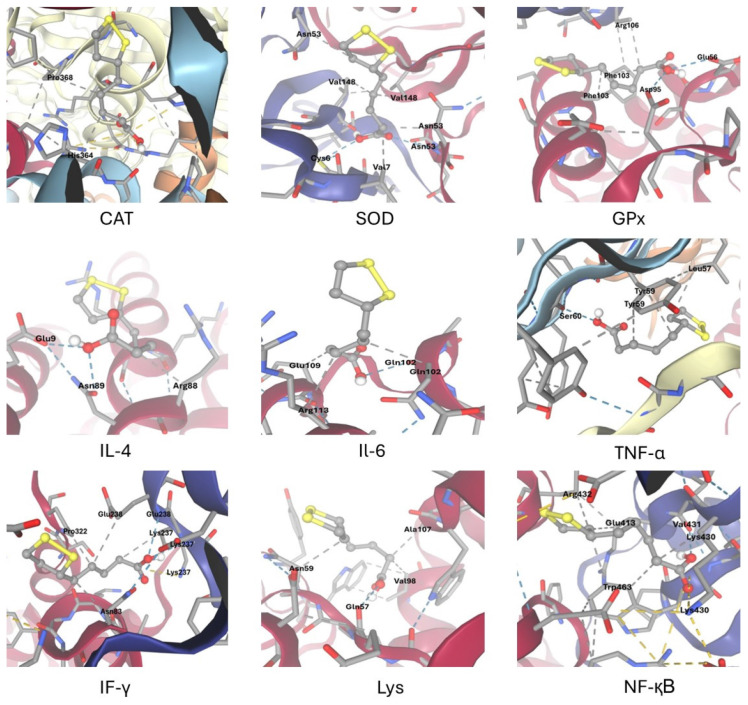
Molecular docking visualization of alpha-lipoic acid (ALA) interactions with selected antioxidant and inflammatory target proteins. CAT, catalase; SOD, superoxide dismutase; GPx, glutathione peroxidase; IL-4, interlukin-4; IL-6, interlukin-6; TNF-α, tumor necrosis factor alpha; IF-γ, interferon gamma; Lys, lysozyme; NF-κB, nuclear factor kappa B.

Molecular docking via SwissDock was used to explore how ALA may protect against OS and inflammation. As shown in Table 3 and Figure 1, molecular docking results demonstrated that ALA strongly interacts with key antioxidant enzymes, including GPx (−5.4 kcal/mol), SOD (−5.0 kcal/mol), and CAT (−4.4 kcal/mol), suggesting its role in enhancing antioxidant defense. ALA also exhibited a high binding affinity for the inflammatory regulator NF-κB (−5.3 kcal/mol), suggesting its potential role in modulating inflammatory responses. Moderate binding affinities were observed with IFN-γ and TNF-α, while Lyz, IL-6, and IL-4 exhibited lower affinities. These findings support ALA’s protective effects against HS by targeting OS and inflammation, especially via GPx and NF-κB. These findings suggest that ALA protects against HS through targeting antioxidant enzymes and modulating inflammatory pathways, especially NF-κB and GPx.

### Characterization of alpha-lipoic acid-loaded chitosan nanoparticles

**Figure 2:**
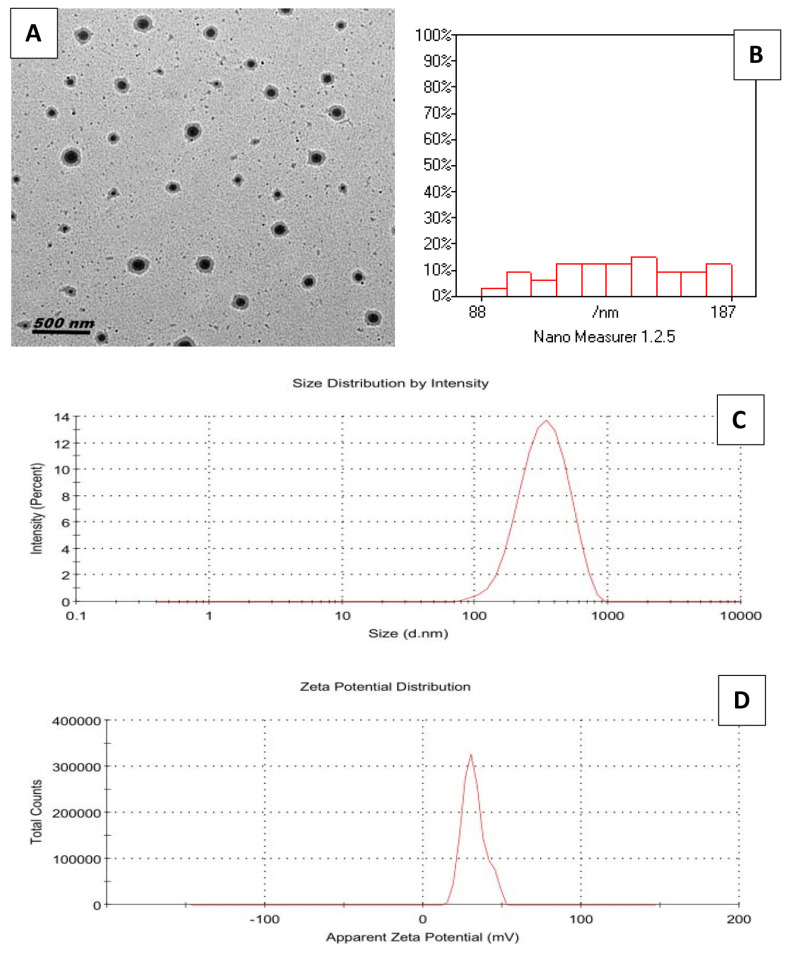
Characterization of alpha-lipoic acid-loaded chitosan nanoparticles. (A) TEM image showing the morphology of ALA-CHNPs, revealing nearly spherical particles, (B) histogram indicates particles are uniformly sized between 88 and 187 nm, (C) zeta size distribution, and (D) zeta potential distribution. TEM, transmission electron microscope; ALA-CHNPs, alpha-lipoic acid-loaded chitosan nanoparticles.

Structural and morphological analyses were conducted to explore the encapsulation efficiency and physicochemical properties of ALA-CHNPs. Figure 2A shows a transmission electron microscopy image of ALA-CHNPs, revealing predominantly spherical particles with minimal tendency to aggregate. The average particle size varied between 88 to 187 nm, as illustrated in Figure 2B. Dynamic light scattering analysis determined a PDI of 0.138 with an average particle size of 298. 4 nm, suggesting uniformity in particle distribution and size (Figure 2C). The ALA-CHNPs show high colloidal stability, as indicated by their positive charge of zeta potential of 31.7 mV, which plays a key role in maintaining particle dispersion (Figure 2D). The LE and LC of ALA in the ALA-CHNPs were calculated to be 78.5%, and 11.3% respectively. These results indicate a high level of ALA encapsulation within the chitosan NPs. These results confirm successful ALA encapsulation and suggest the formulation is stable and potentially bioavailable due to its favorable size, uniform distribution, and surface charge.

### Climatological factors

During the experimental period, the average ambient temperature and RH were 30.84±0.75°C and 73.12±1.08%, respectively, yielding a calculated THI of 29.47±0.43. These data indicated that the rabbits were exposed to severe HS, which justifies assessing ALA-CHNPs for their potential protective action under such conditions.

### Growth performance, feed utilization and physiological response

To evaluate the effect of dietary ALA-CHNPs supplementation on growth performance and physiological adaptation under HS conditions, key indicators such as ADG, FCR, and thermal response were evaluated. Results in Table 4 showed a significant linear increase in ADG with ALA-CHNPs supplementation (p = 0.0324). In contrast, FCR exhibited a linear decrease across all ALA-CHNPs-treated groups compared to the control HS (p = 0.0152), with no significant difference between the ALA-CHNPs200- and ALA-CHNPs400-treated groups. The PI, which indicates the relationship between FCR and LBW, showed a linear increase with ALA-CHNPs supplementation (p = 0.0110). The thermoneutral group had the highest ADG, with no significant differences in PI and FCR compared to the lower-dose ALA-CHNPs groups. With regard to physiological responses, dietary supplementation with ALA-CHNPs significantly reduced both rectal temperature (RT) and respiration rate (RR), with significantly lower values observed in the ALA-CHNPs 200- and ALA-CHNPs 400- treated groups compared to the control HS (p<0.05). The lowest RT and RR were recorded in the thermoneutral group. These findings suggest that ALA-CHNPs supplementation enhances growth, feed efficiency, and HS resilience.

### The carcass traits

Table 5 presents the carcass characteristics. Dietary supplementation with 200 or 400 mg/kg of ALA-CHNPs resulted in a linear increase in both dressing percentage and relative liver weight compared to the control HS (p = 0.0253 and 0.0122, respectively). However, no significant differences were observed between the ALA-CHNPs200- and ALA-CHNPs400 treated groups and the thermoneutral group for either dressing percentage or relative liver weight (p>0.05). In contrast, ALA-CHNPs supplementation did not significantly affect the relative weights of the head, lung, heart, spleen, kidneys, cecum, or intestine compared to the HS (p>0.05). Also, no significant differences (p>0.05) were found in meat composition among the treated groups. These findings suggest that ALA-CHNPs can be used to enhance carcass yield without affecting organ size or meat quality.

### Liver histological investigation

**Figure 3:**
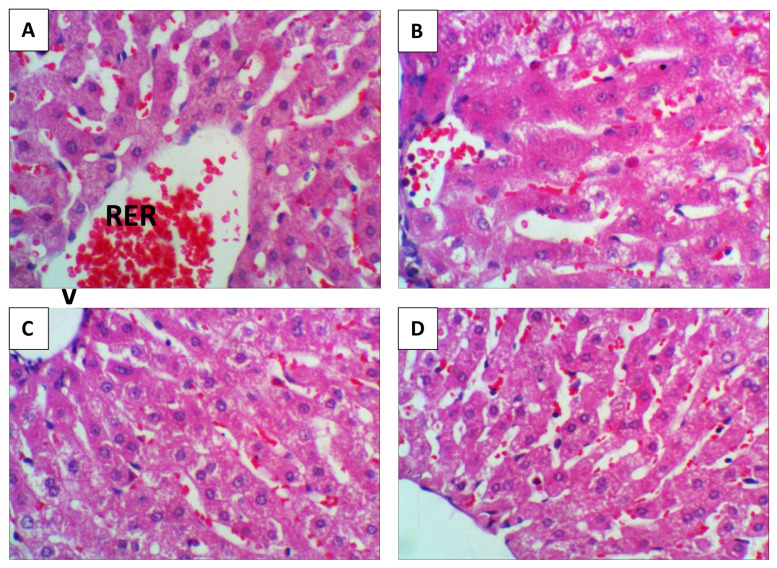
Representative haematoxylin and eosin stained photomicrographs of rabbit liver tissues from the different experimental groups (×400). (A) Group 1 (ALA-CHNPs 0 mg); (B) Group 2 (ALA-CHNPs 100 mg); (C) Group 3 (ALA-CHNPs 200 mg); and (D) Group 4 (ALA-CHNPs 400 mg). ALA-CHNPs, alpha-lipoic acid-loaded chitosan nanoparticles.

**Figure 4:**
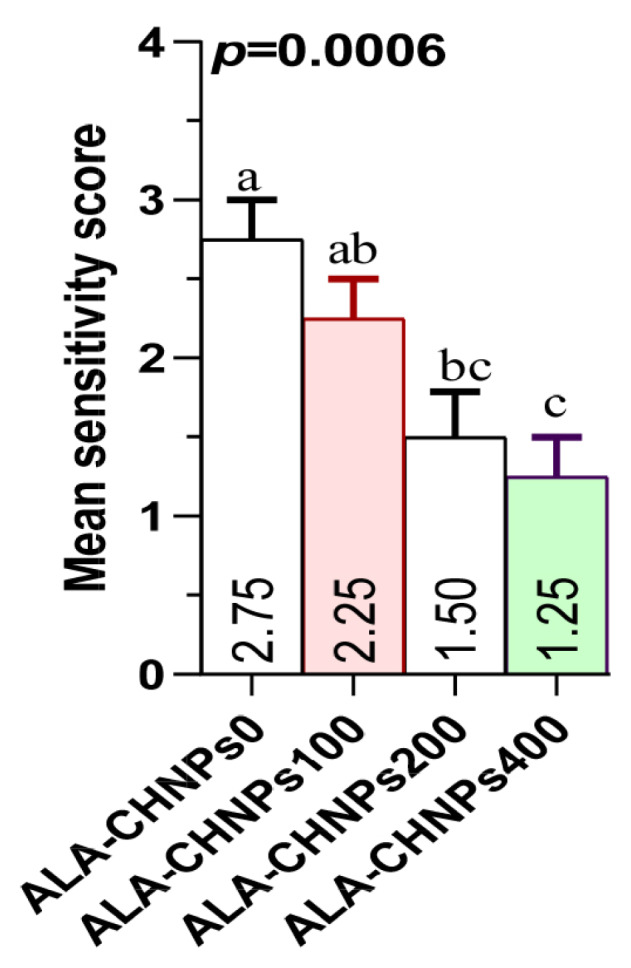
Liver lesion severity scores in heat-stressed rabbits from the control group and those treated with alpha-lipoic acid-loaded chitosan nanoparticles (ALA-CHNPs) at doses 100 mg/kg, 200 mg/kg, and 400 mg/kg.

Figure 3 illustrates the effect of ALA-CHNPs supplementation on histological examination of liver tissue. Photomicrographs of liver sections from rabbits subjected to severe HS and fed diets without antioxidant supplementation showed noticeable histological alterations around the portal vein, characterized by widespread apoptotic hepatocytes, intracellular vacuolation, occasional necrotic nuclei and macrophages, as well as focal perivascular cellular infiltration (Figure 3A). Meanwhile, liver sections from the ALA-CHNPs100-treated group exhibited an almost normal hepatic histological architecture, with only slight microvacuolation detected (Figure 3B). In contrast, the hepato-portal structures of the liver tissue from rabbits in the ALA-CHNPs200- and ALA-CHNPs400-treated groups appeared normal, with hepatic lobules arranged concentrically around the central veins and well- structured hepatic parenchyma. Additionally, the sinusoids remained preserved, with slight cytoplasmic vacuolation detected in the hepatocytes (Figures 3C, 3D). The ALA-CHNPs0 treated group exhibited the highest severity score, followed by the ALA-CHNPs 100-treated group. In contrast, the ALA-CHNPs 200 and ALA-CHNPs 400-treated groups showed the lowest severity scores, with no significant difference observed between them (p>0.05), as illustrated in Figure 4. These histological findings demonstrate that ALA-CHNPs supplementation protects rabbit liver from HS-induced damage.

### Liver ultrastructure

**Figure 5:**
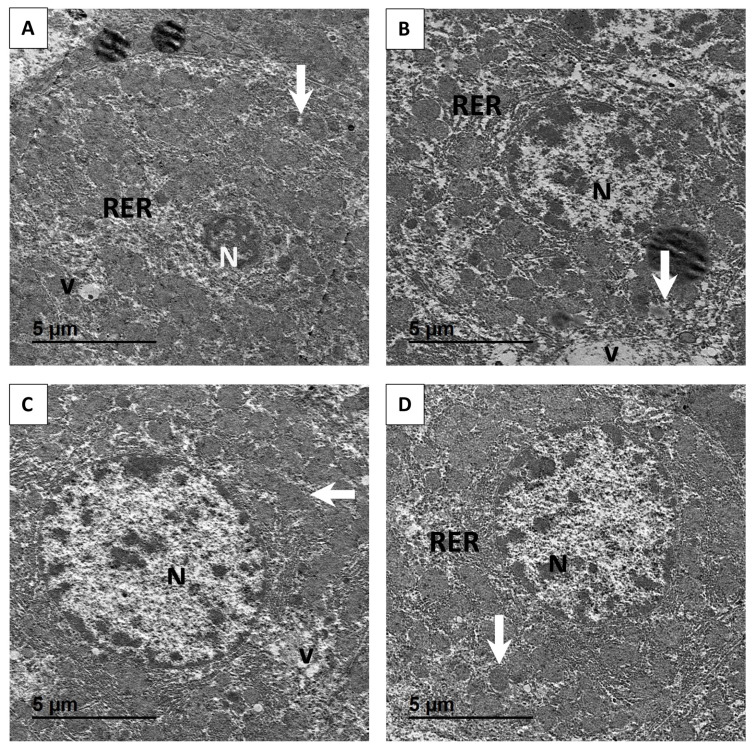
TEM photomicrographs of rabbit liver from different groups. (A) Group 1 (ALA-CHNPs 0 mg); (B) Group 2 (ALA-CHNPs 100 mg); (C) Group 3 (ALA-CHNPs 200 mg); and (D) Group 4 (ALA-CHNPs 400 mg). TEM, transmission electron microscope; ALA-CHNPs, alpha-lipoic acid-loaded chitosan nanoparticles.

Figure 5 demonstrates the effect of ALA-CHNPs supplementation on liver ultrastructure in rabbits subjected to severe HS conditions and fed diets containing varying levels of ALA-CHNPs. The ALA-CHNPs0 group displayed a compact nucleus (N), an enlarged nucleolus cytoplasmic degradation, deteriorated hepatocytes with irregular nuclear morphology, expanded rough endoplasmic reticulum, and multiple vacuolated structures. Moreover, mitochondrial damage was observed, including altered cristae structure, vacuolization, mitochondria with varying shapes, and a reduced mitochondrial count (Figure 5A). In contrast, the ALA-CHNPs 100-treated group exhibited a few degraded mitochondria with mild vacuolization in the cytoplasm (Figure 5B). Similarly, the ALA-CHNPs 200-treated group showed only localized dilation in the rough endoplasmic reticulum, while the cytoplasmic structures remained well-organized. Additionally, the mitochondria and nuclei appeared normal (Figure 5C). The ALA-CHNPs 400- treated group predominantly showed intact mitochondria and cytoplasmic organelles, minimal vacuolization, a well-preserved nucleus, and almost fully intact rough endoplasmic reticulum (Figure 5D). These ultrastructural observations indicate that ALA-CHNPs protect liver cells from HS-induced damage.

### Blood biochemical

**Figure 6:**
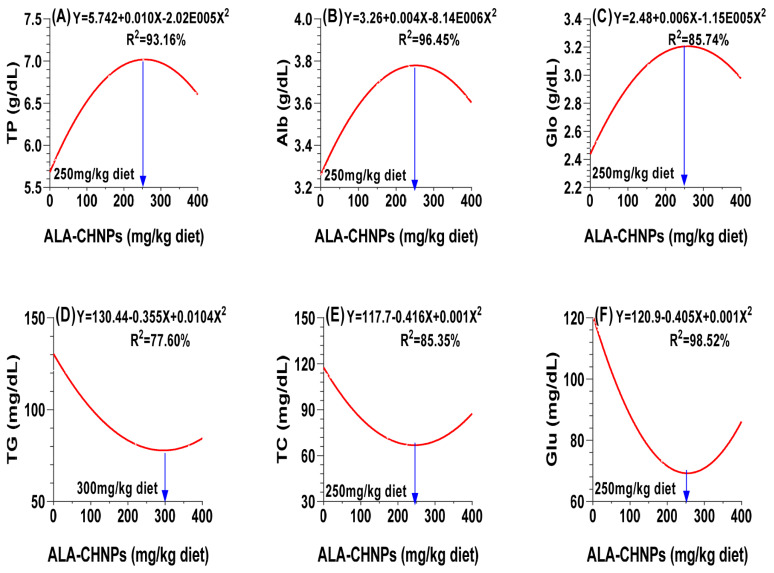
A polynomial regression analysis between dietary levels of ALA-CHNPs and (A) total protein (TP), along with its fractions, including (B) albumin (Alb) and (C) globulin (Glo), as well as the concentrations of (D) blood serum triglycerides (TG), (E) total cholesterol (TC), and (F) glucose (Glu). ALA-CHNPs, alpha-lipoic acid-loaded chitosan nanoparticles.

Table 6 demonstrates that the dietary supplementation with ALA-CHNPs had a significant impact on all blood biochemical parameters (p<0.05 or p<0.0001). The thermoneutral control and ALA-CHNPs200 groups showed no significant differences in biochemical parameters, with the thermoneutral group exhibiting the lowest liver enzyme levels. Serum levels of total protein, albumin, and globulin showed a quadratic increase, peaking at a dose of 250 mg/kg of the diet (Figures 6A–6C). In contrast, the concentrations of TC, TG, and Glu decreased quadratically with ALA-CHNPs supplementation. The optimal doses were identified as 300 mg/kg for TG (Figure 6D), and 150 mg/kg for both TC and Glu (Figures 6E, 6F). Liver function improved significantly in response to the ALA-CHNPs supplementation. The activities of liver enzymes, including ALT, AST, and LDH, as well as total bilirubin levels, decreased in a linear fashion. No significant differences were observed between the ALA-CHNPs200 and ALA-CHNPs400 treated groups. These findings indicate that ALA-CHNPs supplementation improve metabolism and liver function under HS, suggesting their value as a dietary additive to improve animal health and resilience.

### Redox balance

**Figure 7:**
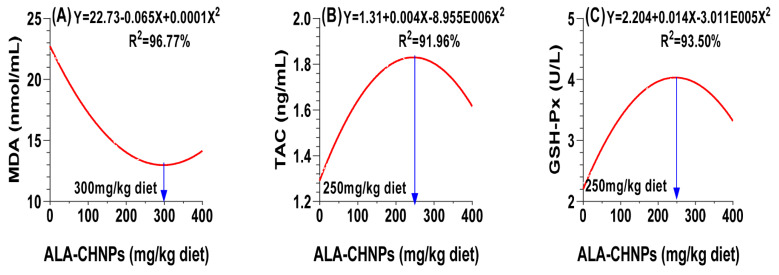
A polynomial regression analysis between dietary levels of ALA-CHNPs and (A) malondialdehyde levels (MDA), (B) total antioxidant capacity (TAC), and (C) the activity of glutathione peroxidase (GSH-Px). ALA-CHNPs, alpha-lipoic acid-loaded chitosan nanoparticles.

Table 7 illustrates the significant positive impacts of dietary ALA-CHNPs supplementation on antioxidant capacity, as indicated by various serum parameters (p<0.05 or p<0.0001). With regard to OS, the addition of ALA-CHNPs to heat-stressed rabbit diets resulted in a quadratic decrease in the lipid peroxidation biomarker, MDA, with the lowest levels observed at a dose of 300 mg/kg of diet (Figure 7A). However, hydrogen peroxide (H_2_O_2_) levels decreased linearly due to the dietary treatment, with no significant differences between the ALA-CHNPs200 and ALA-CHNPs400 groups (p>0.05). Regarding antioxidant enzyme activities, the concentrations of CAT, SOD, and GSH increased linearly in response to the dietary treatment. Additionally, the activity of GSH-Px and the levels of TAC demonstrated a quadratic increase, peaking at a dose of 250 mg/kg of diet (Figures 7B, 7C). The thermoneutral control group showed the highest levels of TAC and antioxidant enzymes, and the lowest levels of OS biomarkers, with no significant differences from the ALA-CHNPs200 group in H_2_O_2_, GSH-Px, and CAT (p>0.05). These results suggest that ALA-CHNPs supplementation boost antioxidants in heat-stressed rabbits, lowering oxidative damage and likely enhancing their health and performance.

### Immunity and inflammatory cytokines

**Figure 8:**
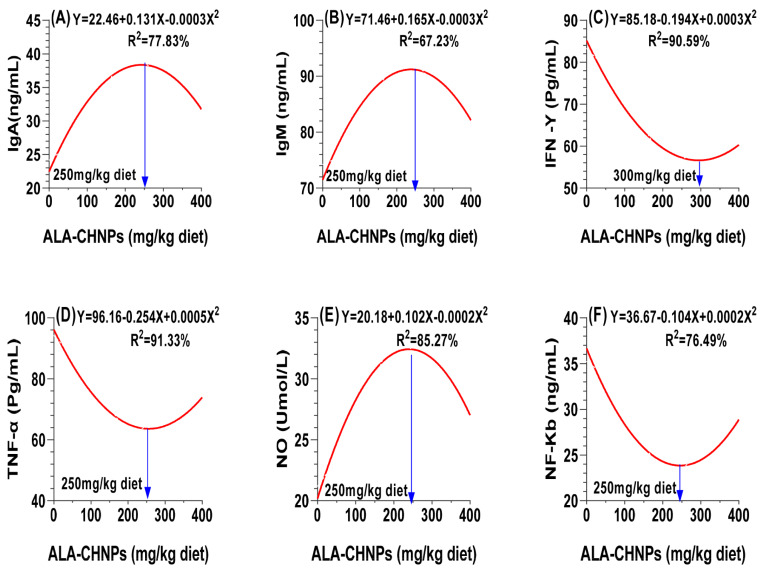
A polynomial regression analysis between dietary levels of ALA-CHNPs and (A) total protein, along with its fractions, including (B) albumin (Alb) and (C) globulin (Glo), as well as the concentrations of (D) blood serum triglycerides (TG), (E) total cholesterol (TC), and (F) glucose (Glu). ALA-CHNPs, alpha-lipoic acid-loaded chitosan nanoparticles.

Regarding immune status, the results presented in Table 8 show that the thermoneutral control group showed the highest levels of immunoglobulins, and Lyz activity, along with the lowest levels of inflammatory cytokines and NF-κB. The dietary supplementation with ALA-CHNPs led to a linear increase in both immunoglobulin G (IgG) levels and Lyz activity. In contrast, the concentrations of IgA and IgM followed a quadratic trend, peaking at a dose of 250 mg/kg diet (Figures 8A, 8B). With respect to inflammatory cytokine responses, IFN-γ and TNF-α levels increased quadratically in response to ALA-CHNPs supplementation, reaching their highest concentrations at 300 mg/kg for IFN-γ and 250 mg/kg for TNF-α (Figures 8C, 8D). Similarly, NO levels showed a quadratic increase, while NF-κB levels decreased quadratically, with both parameters showing optimal responses at 250 mg/kg (Figures 8E, 8F). Polynomial regression analysis revealed a linear relationship between dietary ALA-CHNPs levels and the concentrations of IL-6 and IL-10. However, no significant differences were observed between the ALA-CHNPs200 and ALA-CHNPs400 groups (p>0.05; Table 8). These findings indicate that ALA-CHNPs supplementation enhance immune balance in heat-stressed rabbits, improving resilience.

### Multivariable analyses

**Figure 9:**
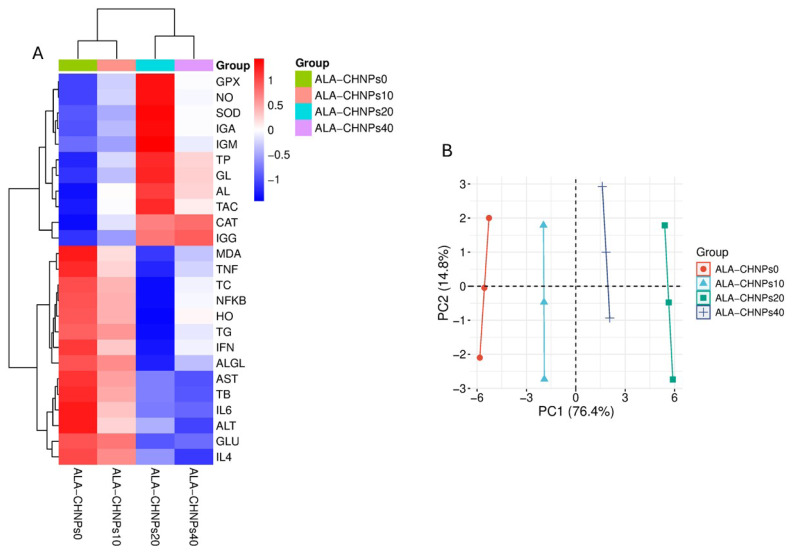
Multivariate analysis of biochemical and immunological responses in heat-stressed rabbits treated with ALA-CHNPs. (A) Heatmap shows dose-dependent up- and downregulation of biomarkers. (B) PCA plot shows distinct group separation with higher ALA doses. ALA-CHNPs, alpha-lipoic acid-loaded chitosan nanoparticles.

Figure 9 presents a multivariate analysis of key biochemical and immunological parameters in heat-stressed developing rabbits treated with different concentrations of ALA–CHNPs. Panel A shows that 400 mg ALA–CHNPs treatment increased antioxidants and decreased inflammatory and stress markers, while the untreated group exhibited low antioxidants and high inflammation and liver stress. Panel B demonstrates clear separation among groups, with the first two principal components (PC1: 76.4%, PC2: 14.8%) capturing most of the data variance. Results indicate dose-dependent physiological and immunological improvements, with the 400 mg ALA–CHNPs group showing the most pronounced therapeutic effect. ALA–CHNPs dose-dependently improved rabbit health; 400 mg showed strongest effect.

## DISCUSSION

High ambient temperatures are becoming an increasingly common challenge in animal farming, particularly for heat-sensitive species like rabbits. HS negatively impacts their health and productivity, mainly through the induction of OS [4]. According to Marai et al [3], the optimal THI for rabbit farming is 27.8. In contrast, the THI in the current study exceeded 31.5, indicating that the growing rabbits were subjected to severe HS. These stressful environments lead to reduced body weight gain, primarily due to decreased feed consumption and disrupted nutrient metabolism [4]. Researchers have increasingly focused on identifying effective alternatives to antibiotics for mitigating the harmful effects of HS, driven by concerns that prolonged antibiotic use could lead to the emergence of antibiotic-resistant pathogenic bacteria [17]. ALA, a medium-chain fatty acid, possesses strong antioxidant and anti-inflammatory properties. Despite its therapeutic potential, its application in industry and pharmacology is constrained by chemical instability, a short biological half-life, and poor water solubility [18]. To address these limitations, encapsulating ALA within polymeric carriers, especially nano-sized particles has been explored as a means to enhance its bioavailability and stability [5]. In this study, ALA-CHNPs were developed and evaluated for their ability to mitigate the negative effects of severe HS in newly weaned rabbits. Physicochemical analysis (size ≈ 298.4 nm; zeta potential ≈ 31.7 mV) indicated good potential for enhanced intestinal absorption and bioavailability. Interestingly, even with increasing amounts of negatively charged ALA, the zeta potential of the NPs remained positive, indicating that chitosan’s positive surface charge remained predominant, implying successful internal encapsulation of ALA within the NP matrix. Dietary supplementation with 200 or 400 mg ALA-CHNP/kg significantly improved ADG and reduced FCR (p<0.05), likely due to enhanced immune function, antioxidant capacity, and more efficient lipid utilization for energy [19]. This will be further discussed in this section. Physiological indicators like RR and RT confirmed that dietary ALA-CHNP supplementation (200 or 400 mg/kg) significantly reduced HS in rabbits (p<0.05). These improvements likely contributed to better growth and feed efficiency, particularly in the 200 mg/kg group. The effect may be linked to enhanced NO synthase activity, promoting vasodilation and improved heat dissipation [20,21].

Despite HS typically reducing blood proteins and impairing cellular immunity due to increased glucocorticoid secretion, which stimulates gluconeogenesis and weakens immune function [3,22], supplementing the diet of growing rabbits with 250 mg ALA-CHNPs per kg is expected to significantly increase blood levels of total protein, albumin, and globulin. This enhancement in blood proteins strengthens immune function, as they play a crucial role in immune defense, including cytokine signaling, the acute-phase response, and the complement system [23]. Kang et al [24] linked the elevated serum protein levels to improved liver function and increased body weight gain, as the liver is responsible for synthesizing proteins. Additionally, ALA has been shown to reduce urinary protein excretion.

Regarding the lipid profile, the present study found a significant increase in TC and TG in the blood serum of rabbits fed an antioxidant-free diet under HS conditions. These conditions led to a notable increase in the mRNA expression of fat synthesis genes and a rise in lipid content in subcutaneous adipose tissue [25], resulting in the buildup of lipids in the liver tissue [4]. Supplementing rabbit diets with 250–300 mg/kg of ALA-CHNPs is expected to reduce lipid accumulation and enhance lipid metabolism. This effect is likely due to ALA’s interaction with PPAR-γ and PPAR-α, key regulators of fatty acid metabolism, which help prevent insulin resistance, hepatic steatosis, and hypertriglyceridemia in animal models [19]. This study confirms the hepatoprotective effects of ALA-CHNPs in heat-stressed rabbits, as shown by reduced liver enzyme activities, lower bilirubin levels, and increased relative liver weight. Histological and ultrastructural analyses revealed that ALA-CHNP supplementation significantly alleviated liver damage, restoring healthier hepatocytes and near-normal liver architecture, consistent with previous findings on the benefits of natural antioxidants [6]. The reduction in serum Glu in rabbits fed diets supplemented with ALA-CHNPs per kg indicates that ALA promotes glycolysis and the conversion of pyruvate to acetyl-CoA, without influencing gluconeogenesis [26].

OS arises when there is an imbalance between the generation of reactive oxygen and/or nitrogen species (ROS/RNS) and the capacity of biological antioxidant defense systems to counteract them, with the imbalance favoring the former [27]. Excessive ROS can lead to the oxidation of lipids, proteins, and nucleic acids. ROS encompasses a variety of molecules, including peroxynitrite, free radicals like hydroxyl (HO), and superoxide (O2), as well as non-radicals such as hydrogen peroxide [28]. Antioxidants are substances that neutralize free radicals, helping to reduce or prevent oxidative damage [29]. Key high molecular weight antioxidant enzymes, such as GSH-Px, and CAT, SOD, and GSH, play vital roles in maintaining the balance between antioxidants and cellular oxidants [29]. Numerous studies have shown that HS adversely impacts the antioxidant defense system [4,6]. HS increases lipid peroxidation and hydrogen peroxide levels while decreasing the activity of antioxidant enzymes such as GSH-Px, CAT, and SOD, due to elevated free radicals and ROS, as observed in the control HS of this study [22]. However, dietary supplementation with 200–300 mg of ALA-CHNPs per kg of diet is expected to enhance antioxidant enzyme activity and reduce the levels of MDA and H_2_O_2_. These findings suggest that ALA-CHNPs helps counteract HS-induced oxidative damage by boosting antioxidant enzyme activities, restoring endogenous antioxidant levels, and effectively scavenging excess ROS [26]. There are limited scientific reports specifically addressing the protective effects of ALA against HS-induced OS in growing rabbits, making direct comparison with our results challenging. However, a growing number of studies have highlighted the antioxidant properties of ALA. It has been shown to prevent and manage various diseases through its antioxidant and anti-inflammatory properties, including its ability to scavenge singlet oxygen, hydroxyl radicals, and hypochlorous acid [18,26].

This study found that while neither HS nor ALA-CHNP supplementation affected mortality rates, HS significantly reduced serum levels of immunoglobulins (IgA, IgM, IgG) and Lyz activity, indicating that high temperatures can impair immune function in fattened rabbits [4]. However, dietary supplementation with ALA-CHNPs significantly elevated serum levels of IgA, IgM, and IgG, along with LYZ activity in heat-stressed rabbits. These results indicate that ALA-CHNPs can effectively mitigate HS-induced inhibition of Lyz activity and immunoglobulin production. This aligns with the findings of Li et al [30] in broilers, who reported a notable increase in immunoglobulin levels supplemented with ALA. Furthermore, the immune function of rabbits is closely associated with the inflammatory responses of their immune organs. Therefore, we next explored the effects of ALA on inflammatory response under HS conditions. Under HS conditions, the rise in IgG and IgM levels in ALA-CHNPs-treated groups may suggest a compensatory immune mechanism rather than direct stimulation of the immune system. Despite their association with disease states, these immunoglobulin increases in this context suggest an adaptive response to heat-induced immune suppression. Enhanced growth performance and health status in the ALA-CHNPs treated groups lend further support to these results. However, due to the multifaceted nature of immune responses under HS, the significance of these immunological findings should be interpreted cautiously.

Previous studies have shown that HS in rabbits significantly increases the levels of inflammatory cytokines, such as IFN-γ, TNF-α, IL-4, IL-6, as reported by Amber et al [31]. Recently, targeting inflammation has become a promising approach to enhance animal health and productivity, while aiding in their adaptation to HS. Anti-inflammatory therapeutic approaches have demonstrated considerable promise in lowering the risk of numerous chronic diseases [32]. In this study, dietary supplementation with 200–250 mg of ALA-CHNPs per kg is expected to significantly reduce serum concentrations of IFN-γ, TNF-α, IL-4, and IL-6 compared to the HS. ALA likely alleviates inflammation by scavenging free radicals, inhibiting inflammatory genes like NF-κB, and promoting the expression of antioxidative genes such as Nuclear erythroid 2-related factor [33]. NF-κB is a critical transcription factor and regulator of immune and inflammatory responses [34]. It can increase the production of inflammatory cytokines by stimulating the activity of cyclooxygenase 2 (COX-2) and inducible NO synthase [35,36]. Therefore, the reduction of NF-κB activity by ALA–CHNPs in this study may decrease the production of pro-inflammatory cytokines, confirming that ALA-CHNPs can suppress the RAGE-NF-κB pathway and potentially reduce the inflammatory response triggered by severe HS [37]. Although NF-κB was measured in serum, it is fundamentally an intracellular transcription factor, and its presence in serum may not accurately indicate its actual activation status. This represents a limitation of this study, and future investigations should utilize tissue-based methods such as Western blotting or RT-PCR for more accurate evaluation. Although excessive NO production is often associated with OS and tissue damage, NO levels in the ALA-CHNPs-treated groups remained comparable to those of the thermoneutral control. These findings suggest that NO may act more as a marker of a regulated immune response rather than a contributor to oxidative injury. Furthermore, the significant reduction in OS markers, accompanied by enhanced activity of key antioxidant enzymes, further supports the role of ALA-CHNPs in mitigating OS under heat exposure. Molecular docking showed strong binding of ALA to antioxidant enzymes (GPx, SOD) and NF-κB, supporting its antioxidant and anti-inflammatory roles. In vivo multivariate analyses confirmed a dose-dependent improvement in antioxidant status and reduction in inflammatory and hepatic stress markers, especially at the highest dose. These findings highlight ALA-CHNPs as a promising nutritional strategy to enhance thermotolerance and systemic resilience in heat-stressed rabbits.

## CONCLUSION

In conclusion, dietary supplementation with ALA-CHNPs, particularly at doses ranging from 200 to 250 mg/kg of diet, can mitigate HS-induced growth retardation, impaired feed utilization, immune dysfunction, disruption of redox balance, blood alterations, and inflammation in fattened rabbits raised in hot climates. These positive effects may be attributed to the anti-inflammatory and antioxidant properties of ALA, as indicated by molecular docking analysis, which revealed varying binding affinities with target proteins associated with inflammatory responses and OS.

## Figures and Tables

**Table 1:** Composition and calculated analysis of the experimental basal diet

Ingredients	g/kg diet
Berseem hay	310
Maize	20
Soybean meal	170
Barley grain	240
Weat bran	205
Limestone	8
Molasses	25
NACL	3
Di-calcium phosphate	14
DL-Methionine	1.5
Premix^1)^	3.5
Total	1,000
Chemical analysis as % on dry matter basis	
Crude protein	17.43
Ether extract	2.27
Organic matter	92.23
Nitrogen-free extract	58.90
Crude fiber	12.28
Ash	9.12

1) Each kilogram contains the following vitamins: Vitamin A (150,000 IU), Vitamin B1 (10 mg), Vitamin B2 (40 mg), Vitamin B6 (15 mg), Vitamin B12 (0.1 mg), Folic acid (10 mg), Vitamin K3 (21 mg), Choline chloride (5,000 mg), Vitamin E (100 mg), Niacin (200 mg), Biotin (0.5 mg), and Pantothenic acid (100 mg). It also contains minerals: manganese (800 mg), zinc (600 mg), copper (40 mg), iodine (500 mg), iron (300 mg), cobalt (100 mg), and selenium (100 mg).

**Table 2:** Standardized grading system for semi-quantitative evaluation of liver tissue lesions

Score	Comprehensive semi-quantitative grading system for hepatic lesion severity
0 (none)	No pathological changes.
1 (mild)	Hepatocellular degeneration and necrosis ranged from rare to mild, with minimal inflammation and vascular congestion observed.
2 (moderate)	Moderate vacuolation; necrosis and inflammation scattered to multifocal; mild vascular congestion.
3 (severe)	Extensive degeneration and necrosis of hepatocytes were evident, with pervasive inflammatory infiltration and moderate to severe congestion within the hepatic vasculature.

**Table 3:** Molecular docking data of alpha-lipoic acid (ALA) with antioxidant and inflammatory proteins involved in oxidative stress and immune response in heat-stressed rabbits

Target proteins	Binding affinity (Kcal/M)	Hydrogen bonds	Hydrophobic contacts	Ionic interaction	Residues
CAT	−4.4	0	2	0	His364, Pro368
SOD	−5	2	5	0	Cys6, Val7, Asn53, Val148,
GPx	−5.4	2	3	0	Glu56, Asn95, Phe103, Arg106
IL-4	−2.9	2	1	0	Glu9, Arg88, Asn89
IL-6	−3.8	1	3	0	Gln102, Glu109, Arg113
TNF-α	−4.5	1	3	0	Leu57, Tyr59, Ser60
IFN-γ	−4.5	1	4	1	Asn83, Lys237, Glu238, Pro322
Lyz	−4.9	1	3	0	Gln57, Asn59, Val98, Ala107
NF-κB	−5.3	1	5	1	Glu413, Lys430, Val431, Trp463

CAT, catalase; SOD, superoxide dismutase; GPx, glutathione peroxidase; IL-4, interleukin4; IL-6, interleukin6; TNF-α, tumor necrosis factor-alpha; IFN-γ, interferon-gamma; Lyz, lysozyme; NF-κB, nuclear factor-kappa B.

**Table 4:** Effects of dietary alpha-lipoic acid-loaded chitosan nanoparticles (ALA-CHNPs) on growth performance, feed utilization, and physiological response in heat-stressed growing rabbits

Items	Treatment (TRT)					SEM	p-values		
	
	TN-CON	ALA-CHNPs0	ALA-CHNPs100	ALA-CHNPs200	ALA-CHNPs400		TRT	L	Q
Growth performance									
IBW (g)	659.13	663.26	665.26	661.26	658.26	10.23	0.8312	0.0961	0.0516
FBW (g)	2,183.14^a^	2,046.45^c^	2,079.93^bc^	2,116.21^ab^	2,117.09^ab^	15.93	0.0294	0.0316	0.3261
ADG (g)	27.21^a^	24.70^c^	25.26^bc^	25.98^b^	26.05^ab^	0.347	0.0324	0.0123	0.1202
PI (%)	44.92^b^	41.68^b^	46.80^ab^	47.25^a^	48.68^a^	1.793	0.0017	0.0110	0.3325
Feed efficiency									
FI (g)	132.26	121.26	112.26	116.37	113.29	10.03	0.3261	0.1974	0.4121
FCR	4.85^a^	4.91^a^	4.44^b^	4.48^b^	4.35^b^	0.059	0.0139	0.0152	0.2415
Physiological response									
RR	125.13^c^	152.15^a^	149.26^a^	137.03^b^	136.83^b^	1.229	0.0008	0.0120	0.3250
RT	37.16^c^	39.21^a^	39.06^a^	38.62^b^	38.41^b^	0.110	0.0003	0.0103	0.1182

CON: thermoneutral control; ALA-CHNPs0, ALA-CHNPs100, ALA-CHNPs200, and ALA-CHNPs400 = 0, 100, 200 and 400 mg alpha-lipoic acid-loaded chitosan nanoparticles/kg diet, respectively.

a–c Means in the same row with different superscript letter following them are significantly different (p<0.05). L and Q are linear and quadratic regression.

SEM, standard error of the mean; IBW, initial body weight; FBW, final body weight; ADG, average daily gain; PI, performance index; FI, feed intake; FCR, feed conversion ratio; RR, respiration rate; RT, rectal temperature.

**Table 5:** Effects of dietary alpha-lipoic acid-loaded chitosan nanoparticles (ALA-CHNPs) on carcass traits in heat-stressed growing rabbits

Items	Treatment (TRT)					SEM	p-values		
	
	TN-CON	ALA-CHNPs0	ALA-CHNPs100	ALA-CHNPs200	ALA-CHNPs400		TRT	L	Q
LBW	2,079	2,035.36	2,069.57	2,043.23	2,066.94	44.63	0.231	0.362	0.117
Carcass traits (as % of live weight)									
Dressing	61.29^a^	55.86^b^	56.61^b^	59.56^a^	60.03^a^	0.294	0.0314	0.0253	0.2108
Head	5.43	5.16	5.16	5.22	5.11	0.152	0.3261	0.2147	0.2396
Liver	3.25^a^	2.86^b^	3.10^a^	3.17^a^	3.20^a^	0.011	0.0004	0.0122	0.3251
Lung	0.58	0.59	0.53	0.61	0.55	0.039	0.3921	0.4514	0.2414
Heart	0.30	0.32	0.33	0.32	0.34	0.021	0.5527	0.3629	0.1944
Spleen	0.08	0.09	0.10	0.08	0.09	0.041	0.2974	0.1088	0.2419
Kidney	0.66	0.63	0.65	0.62	0.64	0.120	0.1532	0.0966	0.0788
Cecum	0.41	0.39	0.41	0.40	0.39	0.141	0.3924	0.3110	0.1528
Intestine	223.26	218.26	220.16	219.38	221.61	3.305	0.5317	0.5125	0.1642
Chemical composition (g/100 g meat)									
Protein	24.36	23.21	22.96	22.95	23.19	0.139	0.4491	0.4623	0.1974
Fat	2.81	2.79	2.84	2.85	2.85	0.284	0.4591	0.2021	0.4493
Ash	1.49	1.42	1.47	1.51	1.48	0.152	0.6291	0.1524	0.1021

TN-CON: thermoneutral control; ALA-CHNPs0, ALA-CHNPs100, ALA-CHNPs200, and ALA-CHNPs400 = 0, 100, 200 and 400 mg alpha-lipoic acid-loaded chitosan nanoparticles/kg diet, respectively.

a,b Means in the same row with different superscript letter following them are significantly different (p<0.05). L and Q are linear and quadratic regression.

SEM, standard error of the mean; LBW, live body weight.

**Table 6:** Effects of dietary alpha-lipoic acid-loaded chitosan nanoparticles (ALA-CHNPs) on blood chemistry in heat-stressed growing rabbits

Items	Treatment (TRT)					SEM	p-values		
	
	TN-CON	ALA-CHNPs0	ALA-CHNPs100	ALA-CHNPs200	ALA-CHNPs400		TRT	L	Q
TP (g/dL)	7.41^a^	5.74^c^	6.34^b^	7.15^a^	6.61^b^	0.153	0.0001	0.0009	0.0051
Alb (g/dL)	4.32^a^	3.26^c^	3.57^b^	3.81^ab^	3.62^b^	0.095	0.0075	0.0094	0.0407
Glo (g/dL)	3.09^b^	2.48^c^	2.77^b^	3.34^a^	2.99^b^	0.077	0.0001	0.0001	0.0316
Alb/Glo	1.40^a^	1.31^a^	1.29^a^	1.21^b^	1.14^c^	0.027	0.0004	0.0006	0.0358
TG (mg/dL)	81.16^c^	130.48^a^	117.16^a^	88.26^c^	106.37^b^	2.234	<0.0001	<0.0001	0.0411
TC (mg/dL)	61.92^c^	117.71^a^	97.34^b^	64.16^c^	88.17^d^	2.451	<0.0001	<0.0001	<0.0001
Glu (mg/dL)	67.11^c^	120.96^a^	90.19^b^	70.18^c^	86.37^b^	1.788	<0.0001	0.4372	<0.0001
AST (IU/L)	16.74^c^	33.29^a^	30.23^a^	24.16^b^	22.93^b^	1.259	0.0001	<0.0001	0.3820
ALT (IU/L)	41.23	68.16^d^	60.28^b^	54.88^c^	50.29^c^	1.663	0.0001	<0.0001	0.3016
TB (mg/dL)	0.42^b^	0.89^a^	0.76^a^	0.55^b^	0.51^b^	0.093	0.0015	0.0004	0.4185
LDH (IU/L)	114.23^d^	188.29^a^	162.35^b^	132.25^c^	127.64^cd^	3.417	<0.0001	<0.0001	0.3624

TN-CON: thermoneutral control; ALA-CHNPs0, ALA-CHNPs100, ALA-CHNPs200, and ALA-CHNPs400 = 0, 100, 200 and 400 mg alpha-lipoic acid-loaded chitosan nanoparticles/kg diet, respectively.

a–d Means in the same row with different superscript letter following them are significantly different (p<0.05). L and Q are linear and quadratic regression.

SEM, standard error of the mean; TP, total protein; Alb, albumin; Glo, globulin; TG, triglyceride; TC, total cholesterol; Glu, glucose; AST, aspartate aminotransferase; ALT, alanine aminotransferase; TB, total bilirubin; LDH, lactate dehydrogenase.

**Table 7:** Effects of dietary alpha-lipoic acid-loaded chitosan nanoparticles (ALA-CHNPs) on redox status in heat-stressed growing rabbits

Items	Treatment (TRT)					SEM	p-values		
	
	TN-CON	ALA-CHNPs0	ALA-CHNPs100	ALA-CHNPs200	ALA-CHNPs400		TRT	L	Q
MDA (nmol/mL)	8.44^d^	22.73^a^	18.26^b^	13.29^c^	14.27^c^	0.741	<0.0001	<0.0001	0.0427
H_2_O_2_ (mM/L)	0.18^c^	0.47^a^	0.42^a^	0.23^bc^	0.22^bc^	0.063	0.0133	0.0012	0.1566
TAC (ng/mL)	2.65^a^	1.31^c^	1.59^bc^	1.85^b^	1.61^bc^	0.073	0.0147	0.0301	0.0298
GSH-Px (U/L)	4.39^a^	2.20^c^	3.13^b^	4.16^a^	3.29^b^	0.219	<0.0001	0.0006	0.0005
GSH (mg/dL)	28.62^a^	11.26^d^	17.25^c^	20.36^bc^	23.33^ab^	1.250	<0.0001	<0.0001	0.6326
CAT (nmol/mL)	0.76^a^	0.21^c^	0.39^bc^	0.52^ab^	0.54^ab^	0.087	0.0037	0.0036	0.2291
SOD (U/mL)	0.88^a^	0.22^c^	0.27^c^	0.47^b^	0.32^bc^	0.061	0.0001	0.0497	0.0726

TN-CON: thermoneutral control; ALA-CHNPs0, ALA-CHNPs100, ALA-CHNPs200, and ALA-CHNPs400 = 0, 100, 200 and 400 mg alpha-lipoic acid-loaded chitosan nanoparticles/kg diet, respectively.

a–d Means in the same row with different superscript letter following them are significantly different (p<0.05). L and Q are linear and quadratic regression.

SEM, standard error of the mean; MDA, malondialdehyde; H_2_O_2_, hydrogen peroxidase; TAC, total antioxidant capacity; GSH-Px glutathione peroxidase; GSH, glutathione; CAT, catalase; SOD, superoxide dismutase.

**Table 8:** Effects of dietary alpha-lipoic acid-loaded chitosan nanoparticles (ALA-CHNPs) on immunity and inflammatory cytokine responses in heat-stressed growing rabbits

Items	Treatment (TRT)					SEM	p-values		
	
	TN-CON	ALA-CHNPs0	ALA-CHNPs100	ALA-CHNPs200	ALA-CHNPs400		TRT	L	Q
IgG (ng/mL)	68.46^b^	49.21^c^	53.23^c^	62.32^a^	63.28^ab^	2.634	<0.0001	0.0014	0.5430
IgA (ng/mL)	45.28^a^	22.46^c^	28.32^b^	41.26^a^	31.20^b^	2.601	<0.0001	0.0002	0.0003
IgM (ng/mL)	98.64^a^	71.46^c^	77.16^bc^	96.26^a^	81.21^b^	2.514	<0.0001	0.0021	0.0020
Lyz (ng/mL)	5.52^a^	2.72^c^	3.95^b^	4.86^a^	4.92^a^	0.425	<0.0001	<0.0001	0.7485
IFN-γ (Pg/mL)	51.26^d^	85.18^a^	74.14^b^	55.74^d^	60.93^c^	1.513	<0.0001	0.0001	0.0003
TNF-α (Pg/mL)	55.93^d^	94.16^a^	81.05^b^	61.19^d^	74.58^c^	2.103	<0.0001	<0.0001	<0.0001
IL-6 (Pg/mL)	71.16^c^	112.24^a^	108.26^a^	94.58^b^	88.89^b^	2.632	<0.0001	<0.0001	0.7218
IL-10 (Pg/mL)	57.16^c^	78.82^a^	71.18^b^	62.25^c^	61.28^c^	1.911	<0.0001	<0.0001	0.0918
NO (Umol/L)	34.39^a^	20.18^c^	25.53^b^	34.14^a^	26.69^b^	1.692	<0.0001	0.0023	0.0017
NF-κB (ng/mL)	16.22^b^	36.67^a^	32.15^a^	21.46^b^	29.35^a^	2.183	<0.0001	0.0108	0.0212

TN-CON: thermoneutral control; ALA-CHNPs0, ALA-CHNPs100, ALA-CHNPs200, and ALA-CHNPs400 = 0, 100, 200 and 400 mg alpha-lipoic acid-loaded chitosan nanoparticles/kg diet, respectively.

a–d Means in the same row with different superscript letter following them are significantly different (p<0.05). L and Q are linear and quadratic regression.

IgG, immunoglobulin G; IgA, immunoglobulin A; IgM, immunoglobulin M; Lyz, lysozyme; IFN -γ, interferon-gamma; TNF-α, tumor necrosis factor-alpha; IL-4, interleukin-4; IL-6, interleukin-6; NO, nitric oxide; NF-κB, nuclear factor *kappa* B.
